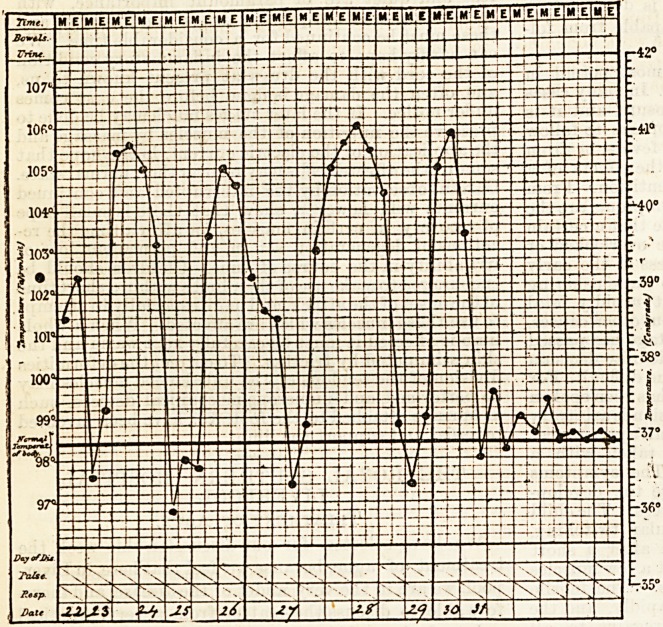# Treatment of Ague

**Published:** 1893-09-23

**Authors:** 


### THE ROYAL SOUTHERN HOSPITAL, LIVERPOOL.

### Treatment of Ague.

We have selected for our discussion this week the treatment of ague, because we feel that here, in Liverpool, we are in the very midst of the disease, and therefore able to discuss the matter from a very practical point of view. Ships leave and enter the port, almost daily, to and from the West Coast of Africa, the hotbed of malaria, and sailors are continually arriving prostrate with fever, so that our wards are never without one or two ague cases. Ague in England is now very rarely heard of, we mean as an endemic disease. This we may venture to suggest is due to the universal better water supply than in former years. That ague is produced by impure water is proved beyond doubt. We might quote the authorities who have brought forward many examples of impure water supply (especially when charged with vegetable matter) producing ague, but we will content ourselves with giving one example, it is reported by Boudin, in "Traité de Geographic et de Statistique Médicales, 1887," and is quoted in Parke's "Practical Hygiene," and runs as follows: "In 1834, 800 soldiers in good health embarked in three vessels to pass from Bona in Algiers to Marseilles. They all arrived at Marseilles the same day. In two vessels there were 680 men, without a single sick man. In the third vessel, the "Argo," there had been 120 men; 13 died during the short passage (time not given), and of the 107 survivors no less than 98 were disembarked with all forms of paludal fevers, and as Boudin himself saw the men, there was no doubt of the diagnosis. The crew of the "Argo" had not a single sick man. All the soldiers had been exposed to the same influences of atmosphere before embarkation. The crew and the soldiers of the "Argo" were exposed to the same atmospheric condition during the voyage; the influence of air seems therefore excluded. There is no notice of the food, but the production of malarious fever from food has never been suggested. The water was, however, different; in the two healthy ships the water was good. The soldiers on board the "Argo" had been supplied with water from a marsh which had a disagreeable taste and odour; the crew of the "Argo" had pure water. This is unquestionably very strong evidence in favour of impure water supply producing ague; but it is the treatment of ague that concerns us now, and must accordingly advance in this direction. Ague is a disease where we see the greatest possible benefit follow the administration of drugs. We refer to sulphate of quinine and arsenic; they act almost as specifics, and are considered as such (especially the former) in malarious districts. The commonest variety of ague—in fact we might say the only variety that we see—is the so-called tertian ague, where the patient has an attack every third day, the temperature intermitting between times. We fortunately never see the remittent variety of ague, for it appears to be a most malignant variety. The accompanying chart shows the temperature in a case of tertian ague, the temperature reaching to over 106 deg. on one of the days.

In discussing the treatment of ague we shall first of all deal with the treatment of the paroxysms, and then go on to the treatment of the actual disease. During the various stages of the paroxysm we endeavour to comfort and relieve the patient by suitable means; for example, during the cold stage, when the unfortunate sufferer is shivering with the cold, we wrap him in blankets surrounded with hot water bottles, and keep up the temperature of the room; hot drinks (such as hot lemonade) are given to assuage the thirst; during the hot stage when he or she is suffering from a hot, dry skin, sponging with tepid vinegar and water gives great comfort. This is about all we can do during the paroxysm of the fever, which fortunately does not last long.

During the intervals of rest we persevere with medicinal treatment, and this we are confident is the more important part of the treatment, and, as one would expect, it varies somewhat. We have (as before noted) in quinine and arsenic very valuable drugs to combat this disease, especially the quinine. Well, then, with regard to the administration of this drug, in some cases it is given as a single large dose before each paroxysm is expected; in others, in small doses at frequent intervals. In the former case we give ten or fifteen grains of the sulphate just before the paroxysm is expected, with the hope that it may be entirely prevented, and if not prevented, at any rate diminished in severity; and certainly in some of the cases it has a marvellous effect. Other cases are treated with the smaller doses of the drug given at frequent intervals. The following mixture is a very favourite one: Quina sulph. gr. iii. acid s. dil. ♏ x. aq. chlorof. ad. ℥i., and this is given every three or four hours, and continued until the patient is convalescent. A few cases do not respond to quinine at all, and these are cases that have been saturated with the drug on the voyage home. It is really astonishing what large quantities of this drug are consumed without any ill effects. The system seems to become accustomed to its use, and large, nay, huge doses can be taken with impunity.

In these cases we prescribe arsenic, and generally prefer the liquor arsenicalis in v ♏ doses every three or four hours, and this generally relieves where the quinine fails. The dose is gradually increased, a careful watch being kept should any untoward symptoms present themselves. By untoward symptoms we mean sneezing, irritation and redness of eyelids, &c. Should such symptoms as these come on the dose is diminished. The medicinal treatment must be continued for some time after all symptoms of the attack have passed away, thereby preventing the disease gaining the ascendancy again.

Aperients, we find, are very necessary during the quinine or arsenic course; the drugs seem to possess more energy for attacking the disease when the evacuations are free and copious. The patient is allowed what he fancies in the way of food, so long as it is nutritious, and can be easily assimilated. Before closing there is one point worthy of notice, and that is the extreme anæmia which ague patients suffer from, especially those who have been subject to the disease for any length of time; it is almost pathognomonic. It is not an anæmia in the strict sense of the word; the skin is more a dusky yellow than the pale transparent skin of anæmia, and they retain this discoloration all through life.

## Figures and Tables

**Figure f1:**